# The Effects of High-Intensity Interval Training on Cognitive and Physical Skills in Basketball and Soccer Players

**DOI:** 10.3390/jfmk9030112

**Published:** 2024-06-27

**Authors:** Sehrish Shiraz, Chiara Salimei, Maurizio Aracri, Carlo Di Lorenzo, Pasquale Farsetti, Attilio Parisi, Ferdinando Iellamo, Giuseppe Caminiti, Marco Alfonso Perrone

**Affiliations:** 1 Department of Clinical Sciences and Translational Medicine, University of Rome Tor Vergata, 00133 Rome, Italy; sehrish.shiraz@students.uniroma2.eu (S.S.); chiara.salimei@gmail.com (C.S.); maurizio.aracri@ptvonline.it (M.A.); carlo.dilorenzo@ptvonline.it (C.D.L.); farsetti@med.uniroma2.it (P.F.); iellamo@uniroma2.it (F.I.); 2 Department of Movement, Human and Health Science, University of Rome Foro Italico, 00135 Rome, Italy; attilio.parisi@uniroma4.it; 3 Department of Human Science and Promotion of Quality of Life, San Raffaele Open University, 00166 Rome, Italy

**Keywords:** Wingate technique, athletic performance, working memory, attention, sprint, agility

## Abstract

As athletes pursue excellence, training techniques continue to advance, making structured physical activity an essential tool for enhancing performance. To optimize athletic performance in modern competitive sports, the balance of physical performance and mental clarity is required. This study seeks to examine the effects of High-Intensity Interval Training (HIIT) on cognitive and physical skills in basketball and soccer players. A 3-week HIIT protocol was incorporated based on the Wingate technique. This study included 10 soccer players and 10 basketball players with an average age of 22.79 ± 1.90 years. Participants performed pre- and post-intervention assessments. Physical proficiency was assessed using 20 m sprint, change-of-direction (COD) and dribbling tests, while cognitive skills were assessed using motion object tracking (MOT), working memory, perceptual load (PL), and attention window (AW) tests. The HIIT intervention significantly improved cognitive performance in particular; noteworthy observations were a 15% improvement in motion object tracking test scores and a 16% increase in working memory test scores for basketball players. The attention window test scores showed a 32% increase, and perceptual load test scores were 31% decreased for soccer players post-intervention. There were significant improvements in physical skills; for example, sprint times were decreased by 6%, and change-of-direction and dribbling times were reduced by 8% and 7%, respectively, indicating improved agility, speed, and ball control abilities. In conclusion, both groups performed significantly better on cognitive and physical skill tests post-HIIT intervention.

## 1. Introduction

Achieving optimal performance in sports is a difficult task that requires a careful balance of physical ability and mental acuity [1]. It takes more than just physical strength to outmaneuver an opponent on the basketball court or make a precise play on the soccer field. Athletes’ ability to think quickly and have highly developed skills are essential for success in these fast-paced sports [2].

Cognitive processes have a considerable impact on athletic performance because they influence decision-making, attention, and motor skill execution when competing [3,4]. Working memory, which retains and analyzes information, is essential for athletes to grasp complex strategic plans and react to changing game conditions in real time [5]. Attentional processes such as attentional control and attention scope influence athletes’ capacity to deploy cognitive resources selectively, sustain concentration in the face of distractions, and successfully monitor critical cues from the environment [6]. Furthermore, perceptual abilities such as the capacity to properly follow moving objects and quickly interpret visual cues are required for athletes to predict opponents’ moves, make timely judgments, and execute precise motor reactions [4,7].

In parallel, physical competence comprises a wide range of motor skills required for athletic prowess, including sprinting speed and agility, as well as ball handling and dribbling ability [7]. Sprinting performance, defined as the maximum velocity reached over short distances, is a critical factor of success in sports that requires fast bursts of acceleration and quick changes in direction [8,9]. Agility, defined as the ability to change direction quickly while retaining velocity and control, is essential for dodging opponents, navigating congested play locations, and performing sophisticated movement sequences with accuracy [10,11]. Furthermore, ball handling talents demonstrated by dribbling in sports such as soccer and basketball necessitate extraordinary coordination, spatial awareness, and refined motor control in order to adeptly handle the ball while dodging defenders and maintaining possession [12].

As athletes strive for greatness, the world of training methods evolves, and High-Intensity Interval Training (HIIT) is one emerging strategy. Knowing the subtle impacts of HIIT on cognitive function is an important endeavor in the quest to maximize athletic performance [13].

HIIT, i.e., the training method, which consists of short bursts of intense exercise followed by brief periods of rest or lower-intensity activity [14], has received attention for its ability to produce significant improvements in cardiovascular health, metabolic function, and muscular strength in a short period of time [13,15].

The literature provides evidence for HIIT’s role in acquisition of skills, which reflects the more complex nature of HIIT towards physiological and neural actions [14]. In fact, the release of neurotrophic factors is associated with synaptic plasticity and neural connectivity leading to cognition improvements [14,16] and serving as the factor that fuels motor learning and acquiring skills [14].

Despite its well-established advantages, there remains room for further research regarding the influence of HIIT on both cognitive and physical performance in athletes, particularly focusing on basketball and soccer players.

Understanding the interdependence of cognitive and physical talents in athletic performance emphasizes the relevance of investigating HIIT’s potential impacts on both domains for players, coaches, and sports scientists alike. While prior studies focused mostly on the physiological changes connected with HIIT, there is a growing interest in investigating its impact on cognitive function and physical skill. The hypothesis tested was that the three-week HIIT program may improve basketball and soccer players’ cognitive and physical skills.

## 2. Methods

An experimental study was conducted in the general preparatory phase for which 10 soccer players and 10 basketball players of average age of 22.79 ± 1.90 years were recruited with at least 5 years of experience in their respective fields of play. Individuals with behavioral issues, learning difficulties, or medical conditions were excluded. Furthermore, the inclusion criteria required participants to be drug-free and had not practiced HIIT for at least six months prior to the commencement of this study. Due to the difficulty of regulating possible variations in estrogen levels, which might affect parameters such as cerebral blood flow and cerebrovascular reactivity, females were purposefully excluded from these investigations, as emphasized by Krause et al. [17].

Adhering to established protocols, the HIIT program employed in this study was adapted from Zwetsloot et al. [18]. To maintain consistency, participants were instructed to abstain from alcohol, caffeine, and intense exercise for 48 h before all tests and HIIT sessions. Furthermore, to reduce any changes in performance related to circadian rhythm, all sessions were held at the same time of day.

The HIIT program used a Monark bicycle ergometer (Monark Ergomedic 824, Varberg, Sweden) with the Wingate system set to 7.5 g per kilogram of the participant’s body weight. Over a span of three weeks, the complete testing procedure comprised a total of nine sessions, with three sessions scheduled on alternate days: Tuesday, Thursday, and Saturday. In the initial week, each session encompassed five sets, during which participants utilized a Monark bicycle ergometer to engage in 30 s of maximal load at peak intensity, followed by a 75 s active rest period at 50 watts. In the second week, the workouts grew more difficult, with eight sets while maintaining the 30 s maximum load and 75 s active rest intervals. In the third and final week of the program, the intensity increased to ten sets with the same maximal load and active rest pattern in each session. Participants performed an active warm-up and cool-down cycling routine for at least three minutes before and after each session, at a cadence of at least sixty pedals per minute.

To look for the effects of HIIT on cognitive and physical skills of athletes, the tests were conducted systematically one day before and one day after the end of the HIIT program.

### 2.1. Cognitive Tests

To generate a suitable setting for focused examination, cognitive tests were administered in a quiet room to reduce possible external distraction. Each testing lasted one hour and included four tests meant to assess various cognitive characteristics. During the cognitive assessments, individuals were positioned at a standardized 45 cm distance from the screen, as an intentional measure. This setup attempted to preserve uniformity in visual circumstances across all participants, which improved the reliability and validity of the cognitive evaluations.

*Motion Object Tracking Test (MOT)*: Based on prior study of Alvarez and Franconeri [19], this study employed a motion object tracking test, in which participants were assigned the task of tracking the positions of a sequence of moving circles presented on a computer screen. The test began with an initial presentation of three blue and four green circles. After three seconds, the blue circles changed to green ones, and the entire group of circles began moving. Throughout the dynamic phase, participants were explicitly encouraged to recall the initial placements of the blue circles. After eight seconds, the circle spinning ceased, prompting participants to locate and identify the original dark-blue circles. The number of circles successfully tracked and recognized was the main variable. This experiment was designed to assess participants’ motion object tracking abilities, especially their ability to focus on specific objects in a dynamic visual field and accurately recall their locations while moving. The use of this procedure increased the evaluation of participants’ ability to track moving objects accurately and precisely.

*Working Memory Test*: To assess working memory, the research methodology incorporated a counting span task, a working memory test [20]. The test required participants to identify and count dark-blue circles in a visually distracting landscape of green circles and dark-blue squares. Following each batch of two to six stimuli, participants were charged with memorizing the count totals and then repeating them in the exact sequence of appearance. Each subject went through fifteen trials, and the scoring system comprised dividing the total correctly recalled stimuli by the maximum feasible score to determine performance. The major goal of this study project was to not only examine the participants’ working memory capacity but also their accuracy in retaining and retrieving information, particularly when challenged with distracting visual components.

*Attention Window Test (AW)*: The test used was inspired by the work of Hüttermann et al. [21]. During each trial, participants were instructed to focus on a central point and count the number of light gray triangles shown at two different distances from the fixation point. Notably, to introduce an element of distraction, light- and dark-gray circles and dark-gray triangles were concurrently displayed during each trial.

The test consisted of a total of 180 trials, with each trial featuring a randomly arranged display of stimuli for a mere 12 milliseconds. Following each trial, participants were required to specify the number of light-gray triangles observed at each designated location. The scoring technique determined the mean of an angular window that included the horizontal, vertical, and diagonal axis and contained valid responses. This approach enabled the evaluation of participants’ attentional focus and object localization abilities in the presence of competing visual inputs. The thorough scope of this exam provided valuable information about the participants’ attention spans and capacity to manage visual distractions.

*Perceptual Load Test (PL)*: The test used was based on the approach provided by Beck and Lavie [22]. Each trial began with a fixation point in the middle of the screen that lasted 1000 milliseconds. The task displays then formed and remained visible for 100 milliseconds. During this brief interval, participants were required to quickly determine and indicate whether the circular field contained the letter ‘X’ or ‘Z’. Participants were expressly instructed to overlook the distracting letter in the center. Answering to “-X-” required one to press the “-C-” key on the keyboard and answering to “-Z-” required one to push the “-N-” key on the keyboard.

The test consisted of 160 trials in which distraction loads varied randomly between high and low levels. The notion was that its outcome could give us information on the level of responsiveness of test subjects to distraction, varying by perceptual load, that would be useful in making decisions about the use of attentional resources under different conditions. Mean response time of accurate responses was the measure of performance evaluation. This provided information on the effects that perceptual load has on athlete performance.

### 2.2. Physical Skill Tests

The effect of HIIT on basketball and soccer players’ physical abilities was evaluated manually using a stopwatch through three different assessments, notably, a sprint test, change-of-direction test, and dribbling test.

*Sprint Test*: In accordance with the widely held belief that athletes in team sports frequently sprint over a distance of 20 m [23], this study purposefully used a 20 m sprint test. The ultimate goal was to determine the players’ sprinting proficiency by recording the amount of time taken to complete the outlined distance. To maintain a high standard of accuracy and to eliminate the effects of the floor surface and wind impact, the test was carried out in a very systematic way indoors on a basketball court which made it possible to create a controlled environment which was good enough for evaluations to be made with precision.

*Change of Direction (COD)*: In sports such as basketball, where players continuously engage in rapid accelerations, decelerations, and sudden changes of direction within confined playing spaces, the ability to execute precise changes of direction (COD) plays a critical role in determining overall performance [11]. The test involved running on a parkour circuit based on the layout of the Illinois agility test. The course was constructed, measuring 10 meters’ length and width of 5 m. Besides the start/finish line, the course was marked with two turning points in addition to the middle and the end. This being added, there were four cones scattered in the middle section while preserving a distance of 3.3 between each of them as it was described in the study [24].

*Dribbling Test:* Dribbling abilities, which include the ability to run, change direction, and maintain ball possession, are considered key skills in the sport and is regarded as one of the most common and influential skills for deciding game outcomes [25]. Participants in this examination were required to navigate a ball through a parkour course designed identical to the Illinois agility test utilized in the COD assessment. The total time taken to successfully complete this course served as the dependent measure, offering valuable insights into the participants’ overall dribbling proficiency before and after HIIT.

This study was approved by the Ethics Committee of the University Hospital Tor Vergata (ID number 41.17) and all the subjects gave their informed consent. This study was conducted in accordance with the Declaration of Helsinki.

## 3. Statistical Analysis

JAMOVI 2.3.28 was used for performing the statistical analysis of the data collected. The normalization of the data was established via Shapiro–Wilk test and all the variables met the assumptions of normal distribution. The independent samples t-test was performed to determine the differences of the pre-test and post-test mean scores within the basketball and soccer players. To know the overall effect of the 3-week HIIT program on both physical skill tests and cognitive tests, paired sample t-tests were used. To achieve the objective, the significance level was set at *p* < 0.05. The results were presented using mean values and standard deviations.

## 4. Results

The paired sample *t*-tests (*n* = 20) provided persuasive evidence of substantial increases in cognitive function across all tests, with *p*-values consistently less than 0.05, as shown in Table 1. The motion object tracking test showed a significant mean difference of −84.35 counted items (t (19) = [−4.21], *p* < 0.001), whereas the working memory test showed a mean difference of −6.45% (t (19) = [−4.01], *p* < 0.001). HIIT significantly affected attention window and perceptual load test scores, with mean changes of −3.22 degrees and 26.90 milliseconds, respectively.

**Figure 1:**
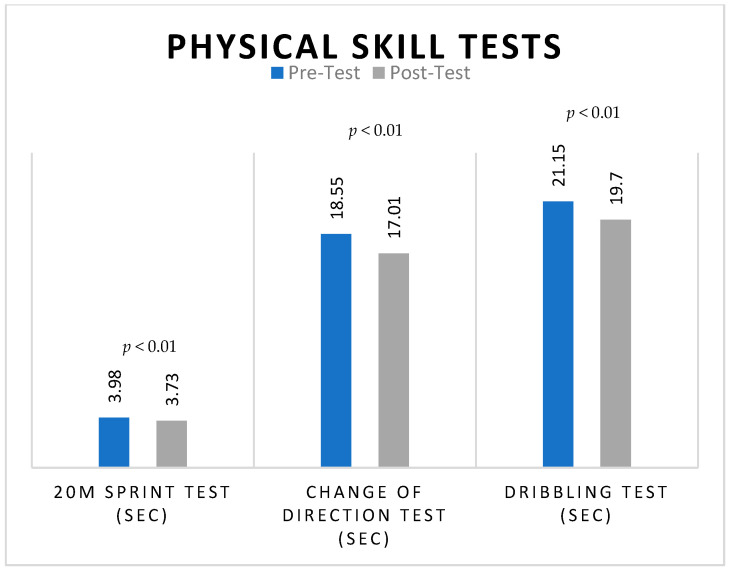
Pre-intervention and post-intervention mean scores of physical skills of basketball and soccer players in total.

In addition to cognitive advantages, this study’s examination of physical skill assessments demonstrated statistically significant changes following the HIIT intervention, as shown in Figure 1. Sprint test timings decreased significantly (pre-intervention: 3.98 ± 0.21; post-intervention: 3.73 ± 3.80), the change-of-direction and dribbling test times were significantly reduced as well, with mean differences of 1.54 and 1.45 s, respectively.

**Figure 2:**
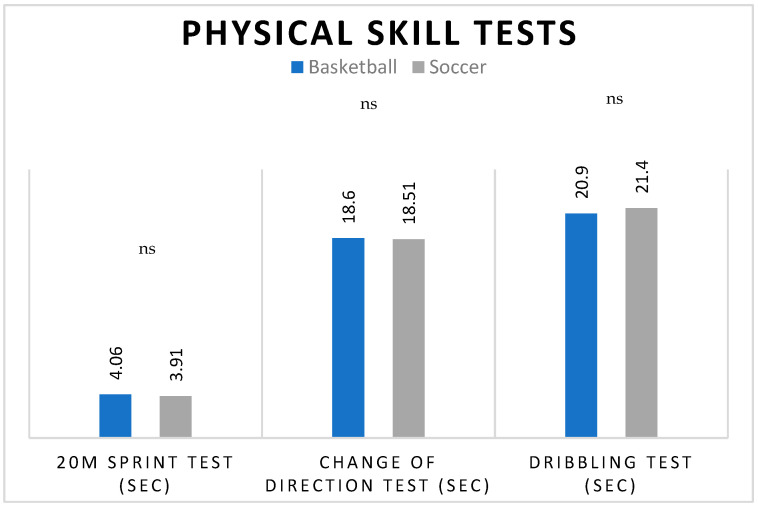
The comparative pre-intervention mean scores of physical skill tests of basketball and soccer players. (ns = not significant).

The independent samples t-tests indicated no statistically significant differences in pre-test results between soccer and basketball players in cognition (Table 2) as well as physical skills (Figure 2), indicating similar baseline performance levels prior to the intervention.

Table 3 shows further statistical analysis concentrating on group-specific data, which clarifies the diverse effects of HIIT. Statistical assessments of cognitive skill tests performed on soccer players provided significant evidence of improvement following the HIIT intervention. The attention window and perceptual load tests showed statistically significant changes in scores (*p* < 0.05), with t-values of −5.96 and −1.91, respectively. However, there were no statistically significant changes to the working memory and motion object tracking test results.

**Figure 3:**
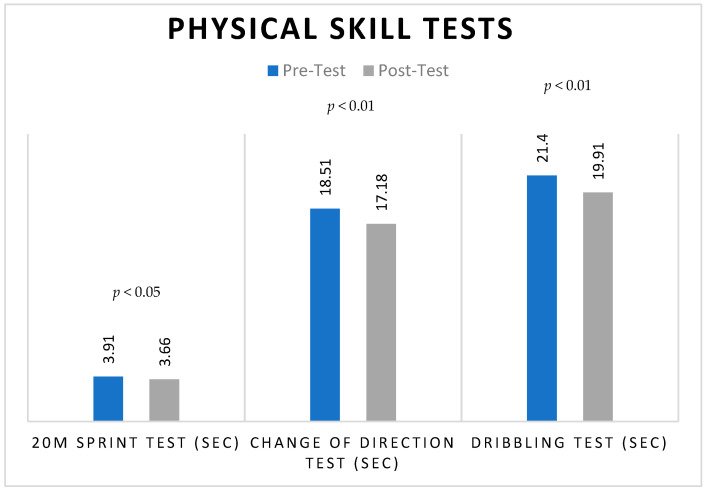
Pre- and post-HIIT training mean scores of physical skill tests of Soccer players.

Apart from that, as showed in Figure 3, significant reductions were observed in the 20 m sprint test (pre-intervention: 3.91 ± 0.17; post-intervention: 3.66 ± 0.35), change-of-direction test time (pre-intervention: 18.51 ± 0.59; post-intervention: 17.18 ± 0.66), and dribbling test time (pre-intervention: 21.40 ± 0.76; post-intervention: 19.91 ± 0.74) of soccer players.

Conversely, the cognitive findings of basketball players exhibited statistically significant improvements in working memory and motion object tracking test scores post-intervention, with *p* < 0.05 and corresponding t-value of −4.28 as shown in Table 4, providing statistical evidence of enhancement. Notably, attention window and perceptual load test scores remained unchanged among basketball players.

**Figure 4:**
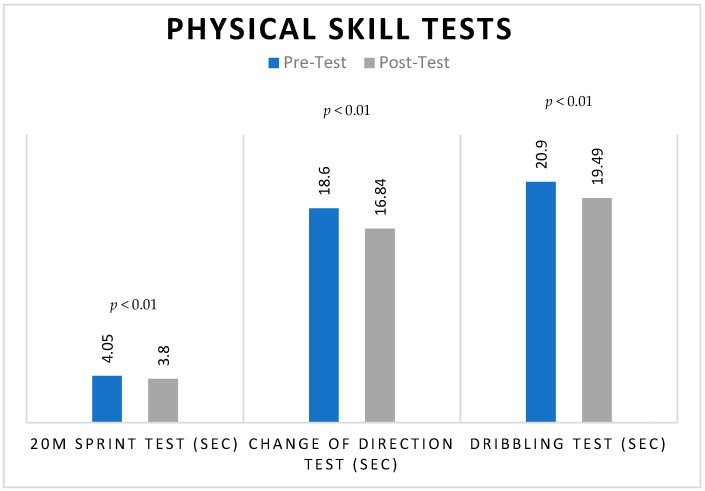
Pre- and post-HIIT training mean scores of physical skill tests of basketball players.

Furthermore, basketball players showed a substantial reduction as well in sprint test time scores (pre-intervention: 4.05 ± 0.23; post-intervention: 3.80 ± 0.16) as presented in Figure 4. Moreover, there were substantial improvements in the time scores of the change-of-direction test and the dribbling test in basketball players, with mean differences between pre- and post-intervention scores of 1.75, and 1.41 s, respectively.

## 5. Discussion

The results of this study that examined the cognitive function and physical performance of soccer and basketball players three weeks post-HIIT produced significant information.

In team sports, coaches and rehabilitation personnel opt for training methods that both decrease musculoskeletal strain and encourage suitable stimuli for both central and peripheral systems. Compared to running-based HIIT, ergometry-based HIIT proves more efficient in increasing lower-limb neuromuscular load and reducing maximal voluntary contraction [26]. Given the emphasis on lower-body strength and flexibility, as well as the need for rapid lateral movements, extensive high jumps, and explosive power in sports like soccer and basketball [27,28], cycling-based HIIT training was selected for this investigation.

Studies have shown an impressive decrease in the time taken to finish sprint tests, change-of-direction tests, and dribbling after HIIT training among soccer and basketball players. This report, in congruence with earlier studies, shows that HIIT training can be used in the training of athletes to raise their agility and speed.

Arslan et al. examined the effect of the running-based HIIT and small-sided game training on basketball players, where it was found that the HIIT team demonstrated better sprint capacity than the other group [29]. Furthermore, the work of Kumari et al. showed that HIIT gave players more agility, as backed by controlled dribbling among basketball players [30]. Soccer players, as observed by Michailidis et al., showed a substantial reduction in sprint test times and improvement in the Illinois agility test performance post-HIIT training [31]. Additionally, Sutharsingh and Kaviraj found significant enhancements in dribbling skills among soccer players following HIIT [32]. The advancements in change-of-direction and dribbling tests highlight the potential of HIIT to augment on-field maneuverability and skill execution, crucial elements for success in dynamic sports such as soccer and basketball.

In addition to the benefits of HIIT for physical performance, this study looked at how it affected cognitive performance in soccer and basketball players. The results revealed significant improvements in cognitive abilities such as working memory and attention windows. These cognitive gains are consistent with prior research by Wei et al. who emphasized the cognitive benefits of HIIT in athletes [33]. Augmented cognitive abilities may boost sports performance by allowing for speedier decision-making, more situational awareness, and better focus during competition.

Furthermore, this study found substantial decreases in reaction time (RT) for both high and low perceptual loads. However, no research on the effect of HIIT on RT in relation to perceptual load in soccer and basketball players were reported. Nonetheless, the study of Saphira et al. on medical students found a substantial reduction in RT after running-based HIIT [34]. Furthermore, research on mountain cyclists by Hebisz et al. reported fewer incorrect reactions and a faster recovery time after combining ergometer-based HIIT and endurance training [35].

Nonetheless, despite the abundance of positive findings, it is crucial to acknowledge the complexities of the results, particularly in terms of MOT measures. While the current study found significant changes in MOT test scores following HIIT interventions, other studies such as the work by Park et al. found no significant improvements in MOT after HIIT in groups of participants participating in various sports, including soccer and basketball [36]. These differences might be due to a variety of factors, including the duration and intensity of the HIIT programs used, individual athlete variability, and the precise characteristics of the MOT tasks used in the trials [34,35,36,37].

This study’s findings have significant implications for athletes, coaches, and sports practitioners. For starters, HIIT training for soccer and basketball players appears to be an excellent way to improve agility, dribbling ability, and cognitive function. Coaches and trainers should design HIIT programs tailored to the unique demands of these sports, with a focus on exercises that simulate game situations and emphasize quick direction changes and decision-making.

Moreover, the cognitive performance improvements demonstrate the similarly deep potential for HIIT to improve not just physical performance but also cognitive processing and decision-making skills across all competitive levels of play. Given the intricate cognitive requirements of soccer and basketball, HIIT can improve cognitive performance and provide players with the competitive advantage they need to better analyze their opponents, grasp tactical situations, and execute flawless passes and shoots even while under duress.

## 6. Limitations of the Study

Despite the significant findings, this study has some limitations that should be considered. The convenience-based sampling strategy resulted in a small sample size. As such, it should be regarded as a “proof of concept” study. Moreover, given the unknown long-term consequences of HIIT, the relatively brief intervention period of three weeks may limit the findings’ broad applicability. Furthermore, only soccer and basketball players were included in the participant pool, which may limit the ability to extrapolate results to other sports. Furthermore, this study’s exclusive focus on male participants highlights the probable inability to extrapolate findings to female athletes. Moreover, the use of a single HIIT procedure and the absence of a control group limit the ability to attribute the observed benefits solely to HIIT.

Additionally, the physical skill tests used in this study largely assess fundamental soccer- or basketball-specific motor skills, such as dribbling and speed, which may not sufficiently account for the complex physical demands of actual gameplay. Similarly, while the change-of-direction test used—i.e., the Illinois agility test—is verified, its weakness is the lack of external stimuli that stimulate real-game scenarios.

## 7. Conclusions

In conclusion, the data show that soccer and basketball players improved significantly in cognitive and physical skills after a three-week HIIT program. While group-specific variances are detected, the overall findings highlight HIIT’s potential as a helpful strategy for improving athletic performance across a variety of sports. The sample size and duration of the training intervention may have impacted the results; further research with bigger samples and longer training durations with follow-ups may yield more substantial insights. Furthermore, looking into other aspects that may impact cognitive and physical performance, such as individual differences, might help us better understand the effects of HIIT on athletic performance.

## Figures and Tables

**Table 1:** Post-intervention cognitive test scores of basketball and soccer players.

Cognitive Skill Tests		Mean	Std. Deviation	Mean Difference	*p*-Value
Motion Object Tracking (N)	Pre-Intervention	662.45	91.96	−84.350	0.0004
	Post-Intervention	746.80	91.43		
Working Memory Test (%)	Pre-Intervention	66.80	8.55	−6.450	0.0007
	Post-Intervention	73.25	7.64		
Perceptual Load Test (ms)	Pre-Intervention	128.95	36.63	26.900	0.0001
	Post-Intervention	102.05	41.97		
Attention Window Test (deg)	Pre-Intervention	16.98	3.33	−3.220	0.0001
	Post-Intervention	20.20	3.22		

**Table 2:** Pre-intervention cognitive test scores of all players.

Pre-Intervention		Mean	Std. Deviation	Mean Difference	*p*-Value
Motion Object Tracking (N)	Soccer	641.70	109.89	−41.500	0.326
	Basketball	683.20	69.44		
Working Memory Test (%)	Soccer	67.80	8.77	2.000	0.615
	Basketball	65.89	8.67		
Perceptual Load Test (ms)	Soccer	124.00	29.27	−9.900	0.557
	Basketball	133.90	43.37		
Attention Window Test (deg)	Soccer	15.74	3.13	−2.481	0.097
	Basketball	18.22	3.21		

**Table 3:** Pre- and post-intervention cognitive test scores of soccer players.

Soccer Players		Mean	Std. Deviation	Mean Difference	*p*-Value
Motion Object Tracking (N)	Pre-Intervention	641.70	109.89	−67.500	0.064
	Post-Intervention	709.20	106.48		
Working Memory Test (%)	Pre-Intervention	67.80	8.77	−2.600	0.088
	Post-Intervention	70.40	7.91		
Perceptual Load Test (ms)	Pre-Intervention	124.00	29.27	38.800	<0.001
	Post-Intervention	85.20	33.76		
Attention Window Test (deg)	Pre-Intervention	15.74	3.13	−5.077	<0.001
	Post-Intervention	20.82	3.63		

**Table 4:** Pre- and post-intervention cognitive test scores of basketball players.

Basketball Players		Mean	Std. Deviation	Mean Difference	*p*-Value
Motion Object Tracking (N)	Pre-Intervention	683.20	69.44	−101.200	0.003
	Post-Intervention	784.40	56.29		
Working Memory Test (%)	Pre-Intervention	65.80	8.67	−10.300	0.002
	Post-Intervention	76.10	6.54		
Perceptual Load Test (ms)	Pre-Intervention	133.90	43.37	15.000	0.080
	Post-Intervention	118.90	44.15		
Attention Window Test (deg)	Pre-Intervention	18.22	3.21	−1.362	0.056
	Post-Intervention	19.58	2.80		

## Data Availability

The data presented in this study are available on request from the corresponding author.
